# Combined Therapy Versus Fortified Anti-VEGF Monotherapy in Type C Polypoidal Choroidal Vasculopathy: Long-Term Outcomes and Exploratory Biomarker Insights †

**DOI:** 10.3390/ijms27031224

**Published:** 2026-01-26

**Authors:** Windsor Wen-Jin Chao, Howard Wen-Haur Chao, Hsiao-Ming Chao

**Affiliations:** 1 Department of Medicine, School of Medicine, Aston University, Birmingham B4 7ET, UK; windsor.chao123@gmail.com (W.W.-J.C.); howard.chao2@nhs.net (H.W.-H.C.); 2 Department of Science, University of British Columbia, Vancouver, BC V6T 1Z4, Canada; 3 Department of Medical Education, Leeds University, Leeds LS2 9JT, UK; 4 Department of Ophthalmology, Shin Kong Wu Ho-Su Memorial Hospital, Taipei 111, Taiwan; 5 Department of Chinese Medicine, School of Chinese Medicine, China Medical University, Taichung 404, Taiwan; 6 Institute of Pharmacology, School of Medicine, National Yang Ming Chiao Tung University, Taipei 112, Taiwan

**Keywords:** PCV, nvAMD, CSCR, anti-VEGF, monotherapy, combined therapy, PDT

## Abstract

While standard anti- vascular endothelial growth factor (VEGF) therapy, with or without photodynamic therapy (PDT), is effective for patients with polypoidal choroidal vasculopathy (PCV), not all achieve optimal visual outcomes. This study aimed to compare fortified (double the dose and the volume of the standard one) anti-VEGF combined with PDT versus fortified anti-VEGF monotherapy and to investigate biomolecular profiles and disease relationships among PCV, neovascular age-related macular degeneration (nvAMD), and central serous chorioretinopathy (CSCR). The goal was to identify novel pathways to inform future therapeutic strategies, including hypoxia-inducible factors (HIF)-1α inhibitors. This retrospective cohort study included 23 eyes with indocyanine green-confirmed type C PCV. One eye treated with transpupillary thermotherapy was not included in the following two groups. Patients received either combined therapy (PDT + fortified-dose anti-VEGF; *n* = 12) or fortified-dose anti-VEGF monotherapy (*n* = 10). Primary outcomes were changes in best-corrected visual acuity (BCVA) and central retinal thickness (CRT). Secondary outcomes included injection burden and recurrence. Exploratory analyses examined aqueous biomarkers, including VEGF, placental growth factor (PlGF), β-catenin, HIF-1α, and Wnt1 across PCV, CSCR, and nvAMD to identify novel therapeutic targets. Significant (*p* = 0.003/*p* = 0.005) median CRT reduction was similar (*p* = 0.468) between groups (combined/monotherapy: 137.5 µm/106.5 µm). BCVA (median [Q1, Q3]) change in logarithm of the minimum angle of resolution (LogMAR) was not statistically significant (*p* = 0.279), with 0.25 [0.00, 0.98] in the combined group versus 0.00 [−0.03, 0.28] in the monotherapy group. Treatment burden of anti-VEGFs per person per year was lower with combined therapy (1.16 ± 0.47# PDT + 2.81 ± 0.92# anti-VEGF injections) compared with monotherapy (4.61 ± 1.49# injections). Six eyes demonstrated recurrence at a mean of 15.5 months. Incomplete regression of polyps and branching vascular networks was observed in all eyes. Exploratory biomarker analysis revealed significantly (*p* < 0.05) higher VEGF and PlGF levels in nvAMD compared with PCV. nvAMD also demonstrated significantly (*p* < 0.05) higher β-catenin and lower HIF-1α levels relative to PCV and CSCR, while no significant biomarker differences were observed between PCV and CSCR. Combined therapy or monotherapy with fortified anti-VEGFs reduced treatment burden and achieved significant anatomical improvement but did not yield superior functional outcomes, highlighting the therapeutic difficulty of type C PCV. Biomarker profiling revealed shared hypoxia-related mechanisms between PCV and CSCR, with elevated HIF-1α compared to nvAMD indicating a “preliminary” possible role for HIF-1α inhibitors. Differential expression of these biomarkers highlights additional molecular pathways that may inform future targeted interventions.

## 1. Introduction

Polypoidal choroidal vasculopathy (PCV) is a distinct choroidal vasculopathy characterized by aneurysmal dilations arising from the inner choroidal vessels beneath the retinal pigment epithelium (RPE). Although initially described as a subtype of neovascular age-related macular degeneration (nvAMD), it is now recognized as a separate clinical entity with unique demographic and angiographic features. Its prevalence varies across ethnicities, accounting for approximately 20–60% of presumed nvAMD in East Asian populations but only 4–10% in White cohorts [1,2,3].

PCV has been classified into different phenotypes based on angiographic characteristics [4]. Type A lesions show interconnecting channels on indocyanine green angiography (ICGA). Type B exhibits a branching vascular network (BVN) with no leakage. In contrast, type C PCV is relatively rare, representing approximately 4–5% of all PCV cases in Caucasian populations and 35% in East Asian populations [1,5]. It is defined by a BVN with late leakage on fluorescein angiography (FA); polyps that tend to be smaller, deeper, or multifocal; and larger vascular complexes that often involve the fovea [6,7]. These anatomical characteristics contribute to poorer visual outcomes and present unique therapeutic challenges, as type C lesions may respond less predictably to standard anti-VEGF monotherapy [4,7].

Current management largely parallels nvAMD treatments [8], primarily involving intravitreal anti-VEGF therapy, either alone or combined with photodynamic therapy (PDT), which reduces exudation and hemorrhage. Optimal regimens, including pro re nata (PRN) versus treat-and-extend, remain under investigation. Advances in multimodal imaging, such as optical coherence tomography-angiography (OCT-A) and spectral domain OCT, have refined lesion characterization, while studies of aqueous biomarkers are beginning to elucidate molecular mechanisms, guiding treatment decisions and potentially reducing treatment burden. Novel anti-VEGF agents (brolucizumab, faricimab) and delivery systems (port delivery) hold potential to further improve outcomes.

Despite these advances, type C PCV remains poorly characterized. Most studies of PCV combine all phenotypes or focus predominantly on type A and B, leaving long-term outcomes and biomarker profiles of type C poorly elucidated. To date, no studies have systematically evaluated outcomes beyond best-corrected visual acuity (BCVA), or examined central retinal thickness (CRT), treatment burden, multimodal imaging, and aqueous humor biomarker profiles specifically in this subtype. Similarly, no published work has explored the therapeutic potential of fortified anti-VEGF therapy, alone or in combination with PDT, in type C PCV.

Understanding the molecular drivers of neovascularization is critical, as treatment response in PCV is largely determined by underlying angiogenic mechanisms. Neovascularization in PCV arises from an imbalance between pro- and anti-angiogenic factors. VEGF-A, potentiated by hypoxia via HIF-1α stabilization, is central to intraocular neovascularization in ischemic retinal diseases, including nvAMD [9,10,11,12,13,14]. Under normoxia, HIF-1α is degraded, but hypoxia stabilizes it, allowing nuclear translocation and transcription of VEGF. Dysregulation of this pathway promotes abnormal vessel growth and leakage, contributing to subretinal edema and retinal cell death. Placental growth factor (PlGF), a member of the VEGF family, synergizes with VEGF-A to amplify angiogenesis. Elevated PlGF is observed in various ischemic retinal and choroidal diseases, driving leakage, inflammation, and endothelial proliferation [15,16]. Its inhibition or downregulation may stabilize vasculature and serve as a biomarker for disease activity or therapeutic response.

Upstream of this, the Wnt1 signaling pathway regulates angiogenesis upstream of HIF-1α and VEGF. Wnt1 ligands bind to frizzled and low-density lipoprotein receptor–related proteins 5 and 6 (LRP5/6) receptors, preventing β-catenin degradation and activating VEGF transcription [8,17,18,19,20,21,22,23,24,25]. Canonical and non-canonical Wnt1 pathways enhance HIF-1α signaling, amplifying VEGF expression under hypoxia, and contribute to upregulation of angiopoietin-2 [26,27,28]. Dysregulation results in fragile vessels prone to hemorrhage and ineffective waste clearance, driving retinal pathology [29]. Despite significant advancements in therapeutic treatments, such as intravitreal anti-VEGF and/or angiopoieitin-2 (e.g., Avastin, Lucentis, Eylea, or Vabysmo), the management of PCV remains a persistent challenge in the field of ophthalmology. Apart from this, it has been accepted that both PCV and central serous chorioretinopathy (CSCR) share pachychoroid characteristics [8], i.e., thick choroids and hyperpermeable vessels, albeit with respective poorer vs. better visual outcomes. The classic subtype of nvAMD was presently included and has been known to involve choroidal neovascularization that originates from the choroid and grows into the macula. To understand these three related identities might be helpful to treat PCV, particularly vision-threatening and poorer prognostic type C.

To address these gaps, we leveraged a comparatively sizeable cohort of 23 eyes with type C PCV to evaluate both clinical and molecular outcomes. We assessed long-term responses to combined therapy (PDT plus fortified anti-VEGF) versus fortified anti-VEGF monotherapy and analyzed aqueous humor biomarkers, including VEGF, PlGF, HIF-1α, β-catenin, and Wnt1. By integrating clinical and molecular insights, this study aimed to clarify the pathophysiology of type C PCV, refine therapeutic strategies, and identify potential molecular targets for this challenging-to-treat subtype.

## 2. Results

### 2.1. Baseline Characteristics

**Figure 1:**
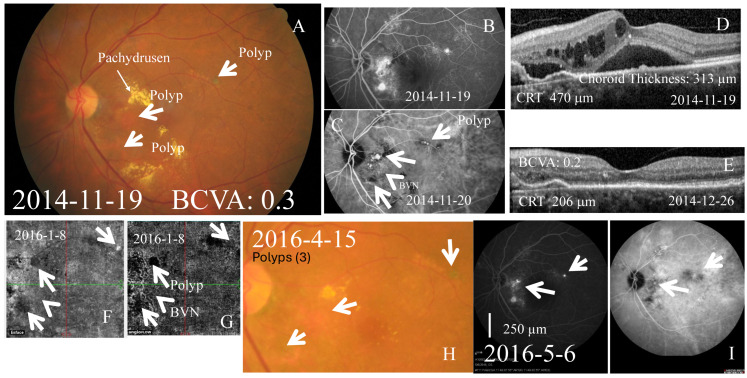
A representative case of type C PCV. Left eye of a 75-year-old female with type C PCV diagnosed on 19 November 2014, color fundus demonstrated salmon-colored polyps (arrows) along papillomacular bundle, temporal upper quadrant, and temporal lower quadrant (**A**). These were further proved by compatible fluorescent angiography (FA; (**B**)) and ICG, namely polyps (arrows; (**C**)) with BVN (arrow head; (**C**) or (**G**)) that present with late leakage in FA (**B**). Simultaneously, OCT revealed cystoid macular edema (CME; (**D**)). One week later, Lucentis was intravitreously injected and CME was relieved by OCT around one month after the defined intravitreous injection (**E**). (**F**) (OCT enface), (**G**) (OCT-A), (**H**) (color fundus photo), and (**I**) (FA, **left**; ICG, **right**), polyps (arrows) were less numerous and less obvious in the presence of BVN (OCT-A in (**G**): as indicated by arrow head). Scale bar = 250 µm.

A total of 23 eyes with indocyanine green-confirmed type C PCV were included. The mean follow-up period was 39.77 ± 7.19 months (Table 1). The mean patient age was 67.78 ± 2.16 years. Representative multimodal imaging of type C PCV is shown in Figure 1. These included salmon-colored (Figure 1A) ICG proved polyps (Figure 1C) with BVN (Figure 1F,G) that presents with late leakage on FA (Figure 1B). One patient (Case 1), who presented with massive subretinal hemorrhage and fluid, was well controlled with TTT (1.5 mm/60 s) and was not classified into the two studied groups

### 2.2. Primary Outcomes: BCVA and CRT

**Figure 2:**
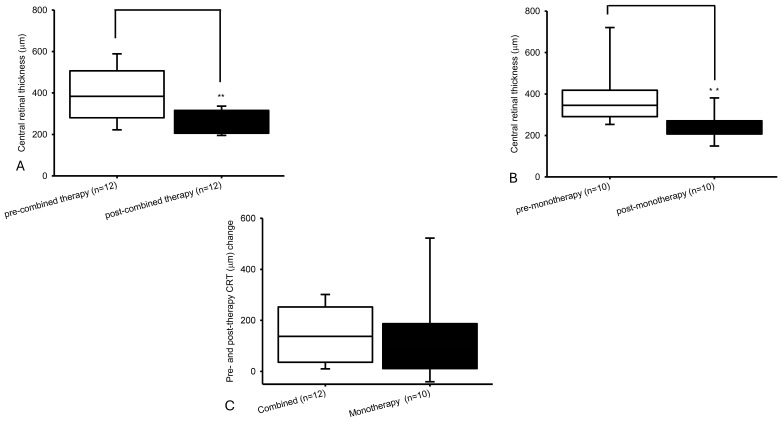
Pre- and post-therapy central retinal thickness (CRT) change. The study regarding the patients with type C PCV showed that Group 1, verteporfin PDT combined with anti-VEGF antibodies (bevacizumab, ranibizumab, aflibercept, or Vabysmo), was compared to Group 2, anti-VEGF monotherapy. The reduction in the CRT was 137.50 [36.00, 252.50] µm vs. 106.50 [11.00, 187.19] µm (**C**). However, the reduction in the CRT was not significantly different (*p* = 0.468). To be specific, following treatment, the CRT was significantly (*p* = 0.003 or *p* = 0.005) reduced in both Group 1 (*n* = 12; from 384.00 [280.50, 506.75] to 243.00 [206.00, 316.00]; (**A**)) and Group 2 (*n* = 10; from 345.00 [291.00, 418.08] to 253.00 [207.25, 270.75]; (**B**)). Furthermore, ** *p* indicated the value of possibility <0.01.

In Group 1 (Figure 2A,C), verteporfin PDT combined with fortified anti-VEGF antibodies (bevacizumab, ranibizumab, aflibercept and/or faricimab) significantly (*p* = 0.003) reduced the CRT from 384.00 [280.50, 506.75] to 243.00 [206.00, 316.00] (median [Q1, Q3]). In Group 2 (Figure 2B,C), anti-VEGF monotherapy (defined antibodies) also significantly (*p* = 0.005) decreased the CRT from 345.00 [291.00, 418.08] to 253.00 [207.25, 270.75]. The magnitude of reduction in CRT was not significantly (*p* = 0.468) different between both groups (137.50 [36.00, 252.50] vs. 106.50 [11.00, 187.19]). As an example, CRT was reduced from 470 μm (Figure 1D) to 206 μm (Figure 1E) one month after one intravitreous injection of fortified Lucentis, accompanied by attenuation of polyps (Figure 1H; Figure 1I, right panel) and fluorescein leakage (Figure 1I, left).

**Figure 3:**
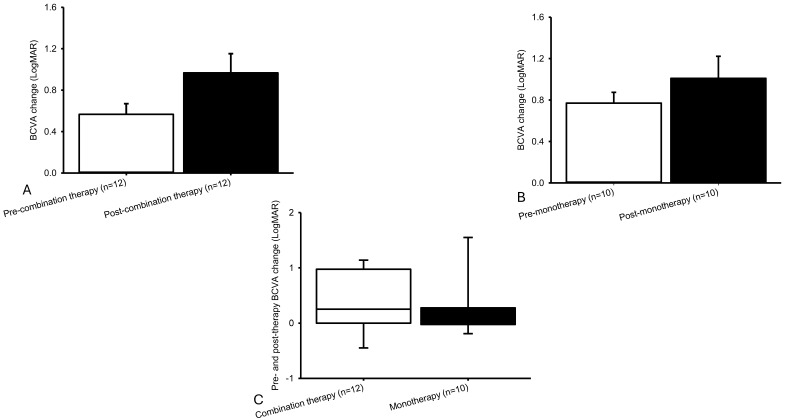
Pre- and post-therapy best-corrected visual acuity (BCVA) change. BCVA changes in LogMAR (**C**) were 0.25 [0.00, 0.98] (combined therapy: from LogMAR 0.57 ± 0.10 to 0.97 ± 0.19; (**A**)) vs. 0.00 [−0.03, 0.28] (monotherapy: from LogMAR 0.77 ± 0.10 to 1.01 ± 0.21; (**B**)). Data were recorded as mean ± SE or median [Q1, Q3].

BCVA change in LogMAR (median [Q1, Q3]; Snellen E: mean ± SE; Figure 3C) was 0.25 [0.00, 0.98] (−0.11 ± 0.07) in Group 1 (Figure 3A) vs. 0.00 [−0.03, 0.28] (−0.01 ± 0.04) in Group 2 (Figure 3B). In Group 1, BCVA in LogMAR (mean ± SE; Snellen E: median [Q1, Q3]) declined, not significantly (*p* = 0.07), from 0.57 ± 0.10 (0.35 [0.10, 0.48]) to 0.97 ± 0.19 (0.20 [0.04, 0.40]). In Group 2, BCVA in LogMAR (mean ± SE; Snellen E with median [Q1, Q3]) decreased minimally and not significantly (*p* = 0.324) from 0.77 ± 0.10 (0.20 [0.09, 0.33]) to 1.01 ± 0.21 (0.15 [0.04, 0.35]). The between-group difference in BCVA change (LogMAR) was not statistically significant (*p* = 0.279).

### 2.3. Secondary Outcomes: Treatment Burden, Recurrence, and Safety

**Figure 4:**
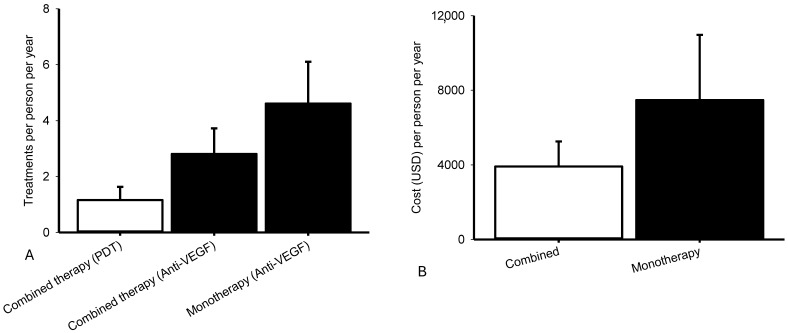
Injection number and cost per person per year. The average injection counts (**A**) were PDT (1.16 ± 0.47#) plus anti-VEGF agents (2.81 ± 0.92#) for combined therapy and anti-VEGF agents (4.61 ± 1.49#) for monotherapy. The mean expense (**B**) was USD 3910.28 ± 1344.46 for combined therapy (PDT plus anti-VEGF) and USD 7471.68 ± 3498.90 for monotherapy (anti-VEGF). “#” means number.

Treatment burden per person per year did not significantly (*p* = 0.34) differ between Group 1 (2.00 [0.36, 3.78]) and Group 2 (2.68 [1.00, 7.53]) (Figure 4A). Eyes receiving combined therapy were treated with an average 1.16 ± 0.47# PDT sessions per person per year and 2.81 ± 0.92# anti-VEGF injections per person per year (=1.12 ± 0.40# Avastin + 1.41 ± 0.50# Lucentis + 0.28 ± 0.21# Eylea). Eyes in the monotherapy group required 4.61 ± 1.49# anti-VEGF injections per person per year (=1.01 ± 0.48# Avastin + 0.42 ± 0.18# Lucentis + 0.23 ± 0.10# Eylea + 2.95 ± 1.69# Vabysmo). Thus, the combined group required fewer anti-VEGF injections overall, although this reduction was offset by the need for 1.16 PDT sessions. Treatment cost per person per year did not significantly (*p* = 0.37) differ between Group 1 (1887.06 [450.95, 7176.33]) and Group 2 (1569.84 [1454.71, 13,078.30]), either (Figure 4B). The mean treatment cost per person per year (Figure 4B) was USD 3910.28 ± 1344.46 for combined therapy compared with USD 7471.68 ± 3498.90 for anti-VEGF monotherapy. Six eyes (26%) developed recurrence at a mean interval of 15.47 ± 4.05 months, and incomplete regression of polyps and BVNs were observed in all cases. No new safety issues were detected, although one patient (Case 13) experienced a reduction in BCVA with LogMAR (Snellen E) from 0.4 (0.40) to 0.8 (0.16) within 3 days of PDT (1.5 mm/83 s) applied to three regions: upper, lower, and medial nasal; 12 May 2016), after which any further PDT was declined.

### 2.4. Exploratory Biomarker Analysis: Various Vascular Markers for PCV, CSCR, and AMD

**Figure 5:**
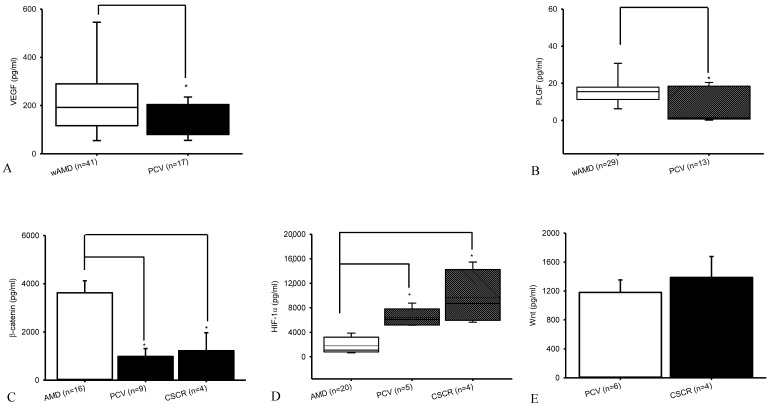
Various vascular markers for type C PCV, CSCR, and nvAMD. Before intravitreous injection (IVI), aqueous protein levels of VEGF/PlGF/HIF-1α/β-catenin/Wnt1 were detected using ELISA on patients with AMD, PCV, or CSCR. In (**A**), the PCV (*n* = 17) or nvAMD (*n* = 41; control) levels of VEGF were median [Q1, Q3]: 106.19 [80.00, 203.81] or 191.91 [116.68, 289.96]. In (**B**), the PCV (*n* = 13) or nvAMD (*n* = 29) levels of PlGF were median [Q1, Q3]: 1.39 [0.72, 18.45] or 15.48 [11.25, 17.90]. The PCV/CSCR/nvAMD (pre-IVI; *n* = 9/4/16) levels of β-catenin (**C**) were 983.77 ± 329.68/1224.33 ± 743.69/3622.41 ± 499.70. The PCV/CSCR/nvAMD (pre-IVI; *n* = 5/4/20) levels of HIF-1α (**D**) were median [Q1, Q3]: 6096.66/8705.06/1091.28 [5173.83/5939.04/803.69, 7811.37/14,239.77/3210.46]. Additionally, the PCV/CSCR (pre-IVI) levels of Wnt1 (**E**) were 1180.66 ± 172.41 (*n* = 6)/1387.12 ± 289.54 (*n* = 4). Significance was * *p* < 0.05.

In a novel approach, prior to intravitreal injection (IVI), aqueous humor protein levels of VEGF, PlGF, HIF-1α, β-catenin, and Wnt1 were measured in patients with nvAMD, PCV, or CSCR using ELISA (Figure 5). VEGF concentrations (Figure 5A) were significantly (*p* = 0.04) higher in nvAMD (*n* = 41; control; 191.91 [116.68, 289.96]) compared with PCV (*n* = 17; 106.19 [80.00, 203.81]). Similarly, PlGF levels (Figure 5B) were significantly (*p* = 0.01) elevated in nvAMD (*n* = 29; 15.48 [11.25, 17.90]) relative to PCV (*n* = 13; 1.39 [0.72, 18.45]).

β-catenin concentrations also differed significantly among the groups (Figure 5C). Mean concentrations were 3622.41 ± 499.70 in nvAMD (*n* = 16), 983.77 ± 329.68 in PCV (*n* = 9), and 1224.33 ± 743.69 in CSCR (*n* = 4). Significant differences were observed between nvAMD and PCV, and between nvAMD and CSCR (*p* < 0.05 for both; Figure 5C).

HIF-1α levels varied substantially across groups (Figure 5D). Concentrations were 1091.28 [803.69, 3210.46] in nvAMD (*n* = 20), 6096.66 [5173.83, 7811.37] in PCV (*n* = 5), and 8705.06 [5939.04, 14,239.77] in CSCR (*n* = 4). HIF-1α levels were significantly higher in both PCV and CSCR compared to nvAMD (*p* < 0.05), suggesting that HIF-1α inhibition could “preliminarily” offer a novel approach for managing PCV. In contrast, Wnt1 levels did not differ significantly (*p* = 0.53) between PCV (1180.66 ± 172.41; *n* = 6) and CSCR (1387.12 ± 289.54; *n* = 4; Figure 5E). These findings indicate disease-specific differences in angiogenic and hypoxia-related biomarkers, supporting further investigation into their mechanistic and therapeutic implications in PCV and related chorioretinal disorders.

## 3. Discussion

In this retrospective cohort study of type C PCV, we observed that combined therapy with verteporfin PDT plus fortified anti-VEGF achieved similar anatomical and functional outcomes compared with fortified anti-VEGF monotherapy, while significantly reducing intravitreal injection burden. Median CRT reduction and BCVA decrease/stabilization did not differ significantly between the two groups, suggesting that combination therapy does not compromise efficacy. While anatomical differences were significant in both groups, BCVA did not significantly change during this “three and a quarter years” (39.77 months) follow-up period. This is consistent with long-term studies of PCV suggesting that despite anatomical improvements, BCVA gains may not always be sustained. For example, Lee and colleagues (2023) demonstrated that BCVA improvement was maintained for three years in eyes showing polyp regression, but visual acuity declined thereafter in persistent cases [30]. Similarly, the EVEREST II trial indicated that anatomical control was better in the combination therapy group, yet long-term BCVA started to decline after two years after combined treatment [31].

However, there are differences to be discussed between our studies. The EVEREST II study evaluated standard-dose anti-VEGF therapy and combined anti-VEGF plus PDT in patients with PCV, without stratifying cases by PCV subtype. In contrast, our study specifically investigated type C PCV, a more treatment-resistant subtype, and employed a fortified anti-VEGF regimen. Although both studies demonstrated anatomical improvement following treatment, the degree and nature of the functional outcomes differed. CRT reductions were comparable, with our monotherapy achieving −106.5 µm and EVEREST II −74.2 µm. However, while post-operative BCVA declined in both cohorts, the decline in our study is comparatively smaller at −0.01 Snellen E compared to −0.11 in the EVEREST II study. The relatively attenuated decline in visual function observed in our study is particularly notable given that type C PCV is harder to treat, whereas EVEREST II included a mixture of PCV subtypes, including less treatment-resistant forms. This suggests that fortified anti-VEGF dosing may provide enhanced functional preservation in more resistant PCV subtypes, highlighting the potential value of tailored, intensified treatment strategies.

Notably, in our cohort, the combined approach required fewer intravitreal injections (2.81 vs. 5.90), although this was partially offset by one or two PDT sessions, resulting in lower overall anti-VEGF treatment costs. These findings align with previous reports showing that PDT can reduce anti-VEGF injection frequency [32,33]. Our findings are consistent with prior studies in PCV demonstrating that combination therapy achieves superior anatomical outcomes and reduced injection burden compared with anti-VEGF monotherapy. Meta-analyses and long-term cohorts have reported greater CRT reduction and higher rates of polyp regression with PDT plus anti-VEGF, though visual gains often remain limited [34,35,36].

While combined therapy can achieve anatomical improvement, visual function does not always correlate, reflecting the progressive nature of PCV and the limitations of current therapies. This motivates investigation into secondary and exploratory outcomes to better understand the biomolecular profile of PCV. The potential roles of argon laser, megadose anti-VEGF (e.g., Eylea 8 mg/0.07 mL), or novel agents such as “hypothesis-generating” HIF-1α inhibitors will be discussed below and warrant further study.

### 3.1. Safety Considerations and Complications

An illustrative case in this study highlights the potential risks and clinical nuances associated with combined therapy. One patient (Case 13) experienced rapid visual deterioration following full-dose PDT (6 mg/m^2^; 1.5 mm/83 s applied to three regions) for PCV with CME, with BCVA in LogMAR (Snellen E) dropping from 0.4 (0.40) to 0.8 (0.16) within three days. Subsequent treatment with fortified Eylea plus Kenacort led to acute endophthalmitis, requiring pars plana vitrectomy. Approximately six months later, the same eye developed retinal detachment, necessitating surgical repair. Despite eight additional intravitreal injections over the following months, BCVA in LogMAR (Snellen E) was only partially restored to 0.7 (0.20) at last follow-up, illustrating how standard PDT therapies can carry substantial risks in complex PCV cases. Caution should be taken that PDT might damage normal retinal neurons and affect visual outcomes. As indicated by Wu and Murphy (1999) [37], PDT uses light-activated drugs and nonthermal light to achieve the selective destruction of choroidal neovascularization with damage on the surrounding normal tissues.

### 3.2. Vabysmo Therapy in Type C PCV with Chronic Pathology: Fibrosis

In three eyes (Case 21, 22, and 23) with type C PCV trialed with fortified Vabysmo over 6.21 ± 2.18 months, CRT decreased modestly but not significantly (Supplementary Figure S2K; *p* = 0.13; 296.3 ± 30.1 µm to 228.0 ± 18.6 µm; mean reduction 68.3 ± 27.4 µm), with complete hemorrhage resolution. BCVA (Supplementary Figure S2L) measured in LogMAR (Snellen E) declined slightly 0.37 ± 0.37, i.e., from 0.73 ± 0.15 to 1.10 ± 0.38, (0.07 ± 0.07); two eyes developed fibrotic subretinal neovascularization (SRNVM), and only two eyes maintained stable vision (0.1 and 0.3). Representative cases showed CRT reductions from 347 to 224 µm (Case 21; Supplementary Figure S2A–D), 299 to 262 µm (Case 22; Supplementary Figure S2E–H), and 243 to 198 µm (Case 23; Supplementary Figure S2I–J). Patients received 6.0 ± 1.7 anti-VEGF injections (mainly Vabysmo, 5.7 ± 2.0) at a mean cost of USD 12,426.7 ± 4425.4. At last follow-up, two eyes (67%) were dry, but vision was limited by SRNVM and fibrosis. These findings hypothetically suggested that dual VEGF/Ang-2 inhibition might stabilize anatomy but could not reverse fibrotic remodeling, highlighting the preliminary thought of early intervention in PCV.

### 3.3. CSCR and Pachychoroid Spectrum: The Relevance to Type C PCV

In our CSCR series (*n* = 4), fortified-dose Vabysmo or Eylea produced significant anatomical improvement. Over 39.6 ± 14.0 months, mean CRT decreased from 358.9 ± 36.8 µm to 210.7 ± 22.3 µm (mean reduction 148.2 ± 25.2 µm; *p* = 0.01), while BCVA in LogMAR with median [Q1, Q3] (Snellen E with mean ± SE) improved from 0.05 [0.00,0.40] (0.78 ± 0.16) to 0.00 [0.00, 0.00] (1.00 ± 0.00), though not statistically significant (*p* = 0.3). Case examples highlight treatment intensity and variable responses: Case 1 received 33 prior anti-VEGF injections (5 Lucentis, 28 Eylea), followed by 8 additional Vabysmo and 8 more Eylea injections, with persistent SRF resolved only after focal argon blue-green laser (VISULAS Green, Zeiss, Jena, Germany) and later responding to megadose Eylea (8 mg/0.07 mL; Supplementary Figure S1A–F). Case 2 achieved complete SRF resolution and marked improvement in metamorphopsia, central scotoma, and contrast sensitivity after a single fortified Eylea injection. Case 3 had persistent SRF after two Lucentis injections, which gradually resolved with three monthly fortified Eylea injections, though it later recurred and was resolved with Vabysmo, accompanied by improvements in visual distortions and contrast sensitivity (Supplementary Figure S1G–K). Case 4 required repeat Vabysmo injection, achieving SRF resolution and full recovery of subjective symptoms at 6 months (Supplementary Figure S1L–O). Across cases, CRT reduction was consistent (Supplementary Figure S1N, upper). Across all four included cases, while BCVA did not reach statistical significance (Supplementary Figure S1N, bottom), subjective contrast sensitivity, central scotoma, and metamorphopsia were markedly improved in all four cases.

Compared with PCV, where anti-VEGF therapy often fails due to fibrotic remodeling, CSCR appears more responsive, suggesting VEGF- and hypoxia-related pathways remain more relevant in this setting. Both conditions share pachychoroid features, including choroidal thickening and vascular hyperpermeability, but their clinical courses differ: CSCR rarely progresses to fibrosis, whereas PCV is prone to hemorrhage and fibrovascular remodeling [38,39], likely explaining the superior efficacy of anti-VEGF in CSCR. Standard-dose regimens may be insufficient in some cases, and dose escalation or mega/fortified doses can enhance receptor saturation and therapeutic effect, as supported by recent studies using double or fortified doses of Eylea and Lucentis in refractory cases [40,41,42,43,44]. Additionally, focal argon blue-green laser may serve as a valuable adjunct for persistent or recurrent subretinal fluid, as demonstrated in some cases (e.g., Case 1).

### 3.4. Biomarker Analyses

As noted above, fortified anti-VEGF therapy, whether administered alone or in combination with other interventions, typically preserves visual function only during the initial years of treatment, with progressive decline observed beyond three years despite optimal management [8,30]. Lim and colleagues have indicated that PCV appears more resistant to anti-VEGF therapy compared with nvAMD, suggesting a distinct pathological pathway [45]. This prompted the present exploratory aqueous biomarker analysis with the aim of providing a mechanistic rationale for these clinical observations. Differential expressions of VEGF, PlGF, HIF-1α, and β-catenin were observed across PCV, CSCR, and nvAMD. Pre-intravitreous injection measurements showed significantly higher VEGF and PlGF levels in nvAMD compared with PCV, consistent with its stronger VEGF dependence and better visual responses typically seen in nvAMD [46,47,48]. β-catenin levels were also higher in nvAMD, whereas HIF-1α was elevated in PCV and CSCR. These findings suggest that CSCR and PCV share a biomolecular profile distinct from nvAMD, supporting the concept of a common pathological precursor. Studies have suggested pachychoroid neovasculopathy as a precursor to both PCV and CSCR [49,50,51]. Interestingly, Wnt1 signaling did not differ significantly between PCV and CSCR, further supporting the hypothesis of a shared pathological precursor.

Overall, these results support a stage-dependent response model: early or non-fibrotic pachychoroid disease, such as CSCR, remains VEGF- and hypoxia-sensitive and responds well to anti-VEGF therapy, whereas PCV, with chronic or fibrotic changes, shows limited functional response despite anatomical improvement. This could also help establish an understanding as to why standard anti-VEGF therapy is unable to improve visual outcomes beyond the first few years of treatment. Elevated HIF-1α in both PCV [52] and CSCR “preliminarily” suggests hypoxia-mediated pathway inhibitors—such as, currently, “hypothesis-generating” HIF-1α inhibitors—may be potential therapeutic targets. Not inconsistently, HIF-1α positive cells were identified in the stroma of PCV samples [52]. CSCR is considered to have lipid deposits at the Bruch’s membrane level to choroid capillaries [53]. These deposits might interrupt the oxygen and nutrients transported to the RPE, and it is highly suspected that HIF/VEGF is expressed in hypoxic RPE. Due to the similar biomolecular profiles, further research could investigate whether CSCR represents a precursor to PCV and whether early treatment reduces PCV incidence [53,54,55].

### 3.5. Limitations and Future Research

While this study provides valuable insights into the outcomes of fortified anti-VEGF therapy in type C PCV, there are some limitations that need to be addressed. Type C PCV is rare, representing only 4–35% of all PCV cases, which makes assembling a large cohort challenging. However, the study design was intentionally focused exclusively on type C PCV to minimize external variables and ensure internal validity. Although the number of patients is modest, this study included 23 patients, making it one of the largest investigating both combined and monotherapy of this uncommon subtype. For comparison, in the EVEREST trial—one of the largest randomized controlled trials—or a large retrospective Netherlands multicenter White cohort study on PCV included only 19 or 15 patients with type C disease when assessing combined therapy and monotherapy across PCV subtypes, respectively [4,5]. In addition, in the series of Yueng et al. [55], 54.5% (*n* = 12) of the total type C PCV patients (*n* = 22) were previously treated. In the present study, 22 patients were fresh type C PCV in contrast to 10 fresh candidates (*n* = 22–12) in the results of Yueng et al. [55]. Moreover, the number of treated patients with BCVA ≧ 20/40 was ≒2 (10.5%) in the EVEREST trial versus 4 (18.18%) in the present study. This is also the first study to investigate the efficacy of fortified anti-VEGF therapy or combination therapy in type C PCV. By focusing on this well-defined patient population, our findings are internally valid and provide a foundation for future research. Despite these limitations, this study provides meaningful insights into the comparative efficacy, treatment burden, and mechanistic underpinnings of type C PCV management. Future research should focus on larger, multicenter prospective studies to confirm our findings and improve their generalizability.

## 4. Materials and Methods

This retrospective cohort study included 23 consecutive eyes with treatment-naïve type C PCV, characterized by polyps and BVNs with leakage on fluorescein angiography (FA) [4]. All cases were confirmed by indocyanine green angiography (ICGA) and multimodal imaging (color fundus photography, FA, and OCT). One eye administered with transpupillary thermotherapy (TTT; IRIDEX Corporation, Mountain View, CA, USA) was excluded from the following two groups: patients received either combination therapy (Group 1: PDT + intravitreal anti-VEGF; *n* = 12) or anti-VEGF monotherapy (Group 2; *n* = 10). To avoid endophthalmitis, full-dose PDT (Verteporfin 6 mg/m^2^; 83 s; Alcami Carolinas Corporation, Charleston, SC, USA) was given, 3 days following anti-VEGF injection, with a strict reminding of sun protection. Anti-VEGF therapy included fortified doses of Avastin (bevacizumab; 0.25 mg/0.1 mL; Genentech, Inc, South San Francisco, CA, USA), Vabysmo (faricimab; 12 mg/0.1 mL; Hoffmann-La Roche Ltd, Wurmisweg, CH-4303 Kaiseraugust, Switzerland), Eylea (aflibercept; 4 mg/0.1 mL; Bayer AG, Bloomington, IN, USA), or Lucentis (ranibizumab; 1 mg/0.1 mL; Novartis AG, Basel, Canton of Basel-Stadt., Switzerland). “Fortified” was defined as both double the dose and double the volume as compared to the standard one, i.e., Avastin (0.125 mg/0.05 mL), Vabysmo (6 mg/0.05 mL), Eylea (2 mg/0.05 mL), or Lucentis (0.5 mg/0.05 mL). Fortified dosing aimed to maximize VEGF suppression and reduce treatment burden, informed by emerging evidence that high-dose aflibercept (8 mg) in nvAMD improves anatomical outcomes and extends durability compared with standard dosing [40,41,42]. For ranibizumab, prior phase I/II studies in PCV reported that 1.0 mg was safe and provided anatomical benefit [43,44]. The rationale for fortified faricimab was extrapolated from these high-dose aflibercept and ranibizumab studies, supported by the AU2022275786A1 patent formulation range (100–400 mg/mL) and emerging evidence of anatomical benefit in treatment-resistant eyes [56]. This strategy aimed to achieve more complete VEGF inhibition in cases potentially less responsive to standard dosing.

Following the manufacturer’s instructions, a filter needle on a 1 mL syringe was used to collect >0.1 mL of the specified antibodies. An insulin syringe (BD Ultra-Fine II, Holdrege, NE, USA; 0.5 mL) delivered 0.1 mL of anti-VEGF for IVI. Prior to IVI, 0.1 mL of aqueous humor was withdrawn via a new 1 mL syringe for biomarker analysis. Pre-/post-IVI intraocular pressures measured by air tonometry (TonoVue-P, Crystalvue Medical, Taoyuan, Taiwan) were 16.51 ± 0.82 mmHg and 17.41 ± 0.87 mmHg, respectively (*p* = 0.45).

### 4.1. Study Outcomes

The primary outcome was change in BCVA (LogMAR and Snellen E) and CRT. Secondary outcomes included treatment burden (number of anti-VEGF injections and PDT sessions), treatment cost, and disease recurrence. Exploratory analyses evaluated aqueous humor samples from all participants to evaluate molecular mediators potentially involved in the pathophysiology of PCV, including VEGF, PlGF, β-catenin, HIF-1α, and Wnt1. These analyses were designed to identify potential therapeutic pathways/biomarkers that could inform treatment strategies.

### 4.2. Ethics

All participants provided written informed consent. This study adhered to the Declaration of Helsinki and was approved by the institutional ethics board of Cheng Hsin General Hospital (CHGH) for both retrospective review and biomarker collection [CHGH-IRB (642) 107-14 and (439) 103-16], as part of the Phenotyping Asian Age-Related Macular Degeneration study. Multimodal imaging, including color fundus photography (Zeiss Clarus 500, Jena, Germany), fluorescein angiography (Heidelberg HRA, Heidelberg, Germany), ICGA (HRA, Heidelberg, Germany), and OCT (Zeiss Cirrus 5000 HD-OCT, Jena, Germany), was performed in the same session to ensure comprehensive baseline evaluation.

### 4.3. Enzyme-Linked Immunosorbent Assay (ELISA)

Protein levels in aqueous humor were quantified using commercial ELISA kits [21,57]: VEGF (BMS227/2, Bender MedSystems, Wien, Austria), PlGF (CBS-E07400r, Cusabio, Houston, TX 77054, USA), HIF-1α (M00013-2, Uscn Life Science, San Jose, CA 95123, USA), β-catenin (DYC1329, R&D Systems, Minneapolis, MN 55413, USA), and Wnt1 (RHF128CK, Antigenix America, Huntington Station, NY 11746, USA) [57]. Total protein concentration was measured using a bicinchoninic acid (BCA) assay (Thermo Fisher Scientific, Waltham, Massachusetts, USA) [58]. ELISAs were performed according to the manufacturers’ protocols. Briefly, aqueous samples (1:5 dilution) and serially diluted standards (0–1000 pg/mL) were incubated in antibody-coated 96-well plates, followed by detection with biotin-conjugated antibodies and HRP/streptavidin. Colorimetric reaction was developed with TMB substrate, stopped with 2N H_2_SO_4_, and optical density was read at 450 nm using a spectrophotometer (ELx800, Biotek, Shoreline, WA 98133, USA). Protein concentrations were calculated from standard curves and expressed relative to controls (normalized to 100%).

### 4.4. Statistical Analysis

Graphs were plotted with SigmaPlot 12.5 (Systat Software, Inc., San Jose, CA, USA; analyses used SPSS 20 (Developer: IBM Corporation, Armonk, NY, USA). Normality was tested (Kolmogorov–Smirnov, Levene) [59]. Two-group comparisons used Student’s *t*-test; multi-group comparisons used ANOVA or nonparametric ANOVA. Data are expressed as mean ± SE or median [Q1,Q3]. Significance was * *p* < 0.05. Furthermore, ** *p*, or *** *p* respectively indicated the value of possibility < 0.01, or < 0.001.

## 5. Conclusions

In conclusion, a fortified anti-VEGF regimen, particularly when combined with verteporfin PDT, effectively reduces CRT and helps stabilize BCVA in patients with type C PCV. Despite these anatomical gains, functional outcomes, as measured by BCVA, remained stable or showed minimal change, indicating that visual improvement did not always parallel structural improvement, and vision may continue to decline over the first few years of treatment despite optimal therapy. Type C PCV remains relatively resistant to conventional anti-VEGF therapy, a finding supported by our biomolecular profiling showing significantly lower VEGF levels compared with nvAMD, and proposing a similar biomolecular profile to CSCR. Further studies are needed to determine whether early intervention in CSCR, and the use of transpupillary thermotherapy, argon laser, fortified (or megadosed) anti-VEGF, or HIF-1α inhibitors, can reduce progression to PCV and improve long-term visual outcomes.

## Figures and Tables

**Table 1:** Baseline characteristics of Combined therapy vs. Monotherapy group.

PCV	Combined Therapy (*n* = 12) §	Monotherapy (*n* = 10)	*p*
Age (y-o)	67.17 ± 3.09	67.60 ± 3.35	0.93
Gender	10♂ + 2♀	7♂ + 3♀	0.44
F-U (month) §§	32.00 [24.75, 84.25]	29.60 [9.00, 32.48]	0.25
BCVA (LogMAR)	0.40 [0.33, 1.00]	0.70 [0.48, 1.08]	0.17
CRT (μm)	396.50 ± 36.29	382.31 ± 45.21	0.81
GLD of lesions (mm)	3.08 ± 0.31	3.37 ± 0.66	0.68
Polyp No.	1.50 [1.00, 3.00]	1.50 [1.00, 2.25]	0.57

The values were expressed as mean ± SE or median [Q1, Q3]. Abbreviations: PCV, polypoidal choroidal vasculopathy; F-U, follow-up; BCVA, best corrected visual acuity; LogMAR, Logarithm of the Minimum Angle of Resolution; CRT, central retinal thickness; GLD, greatest linear diameter, GLD. § As follows, the inside-the-parenthesis PDT No. for each of 12 cases was illustrated in note of this Table: Case 2 (*n* = 1); Case 3 (*n* = 2); Case 4 (*n* = 2); Case 5 (*n* = 2); Case 9 (*n* = 1); Case 10 (*n* = 1); Case 11 (*n* = 1); Case 12 (*n* = 3); Case 13 (*n* = 1); Case 14 (*n* = 3); Case 15 (*n* = 1); Case 16 (*n* = 1). To avoid endophthalmitis, full dose PDT (Verteporfin 6 mg/m^2^; 83 s) was given, 3 days following anti-VEGF injection, with a strict reminding of sun protection. §§ The mean follow-up period was 39.77 ± 7.19 months.

## Data Availability

The original contributions presented in this study are included in the article/Supplementary Materials. Further inquiries can be directed to the corresponding author.

## Supplementary Materials

The following supporting information can be downloaded at: https://www.mdpi.com/article/10.3390/ijms27031224/s1.
