# Supplementary material for: Combined Therapy Versus Fortified Anti-VEGF Monotherapy in Type C Polypoidal Choroidal Vasculopathy: Long-Term Outcomes and Exploratory Biomarker Insights
Source: Int J Mol Sci. 2026 Jan 26;27(3):1224. doi: 10.3390/ijms27031224 (PMC12897784; doi:10.3390/ijms27031224)
Supplement: Supplementary file 1 [file ijms-27-01224-s001.zip › ijms-3960308-supplementary.pdf]

Supplementary Figure S1

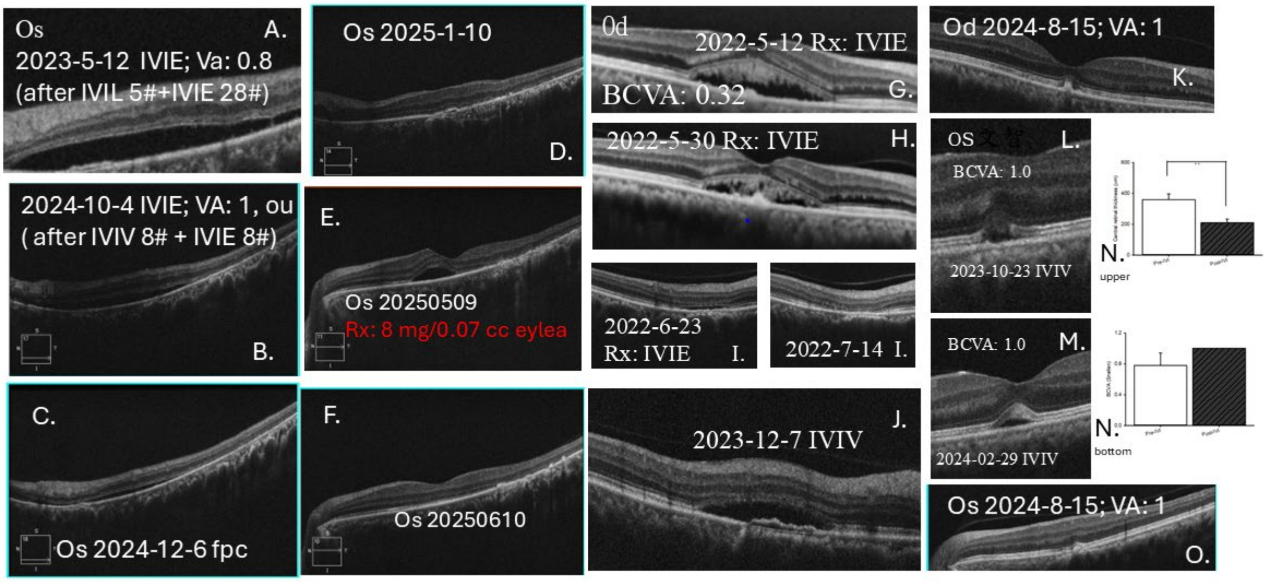

Supplementary Figure S1. CSCR (n = 4) treated with fortified Vabysmo and Case 1 with residual SRF at the lower retina. Case 1 of CSCR received administration with 49 injections of anti-VEGFs (5# fortified Lucentis + 36# fortified Eylea + 8# fortified Vabysmo); specifically, 5# fortified Lucentis plus 28# fortified Eylea did not well control the victim's SRF on May 12th 2023 (A), fortified Vabysmo was initially injected on June 9th 2023 (B) and continually injected for each of another 7 visits (last one on July 5th 2024). Until October 4th 2024, a further 8 injections of fortified Eylea were prescribed with still persistent lower SRF (C), which was dramatically and completely healed by focal argon blur green laser (D). Unfortunately, there was recurrent lower SRF (E), which was again surprisingly and completely healed by megadose of Eylea (8 mg/0.07 cc; F).

In case 3, after three intravitreous injections of Lucentis (0.5 mg/0.05 cc) at one medical center, CSCR did not respond with persistent SRF (G). After three injections of fortified Eylea (4 mg/0.1 cc; Figure S1H and I left), SRF was obviously fading with IS-OS disruption (I right). Unfortunately, CRCR recurred with obvious SRF (on 2023-12-7; J). SRF was responsive to 3 intravitreous injections of fortified Vabysmo and effectively absorbed with best-corrected vision of 1.0 on August 15th 2024 (K).

Four cases of CSCR with subretinal fluid, which was treated by intravitreous injections of Vabysmo with significant central retinal thickness, CRT, reduction (N upper). However, BCVA was improved but not significant (N bottom). For example, in case 4, the left eye had CSCR with sensory detachment (L; M), which was relieved by 3 intravitreous injections of Vabysmo with absence of SRF (O). Abbreviations: central chorioretinopathy (CSCR), subretinal fluid (SRF), intravitreous injection of Vabysmo/Eylea/Lucentis (IVIV/IVIE/IVIL); best corrected visual acuity, BCVA; Rx: treatment; focal photocoagulation, fpc.

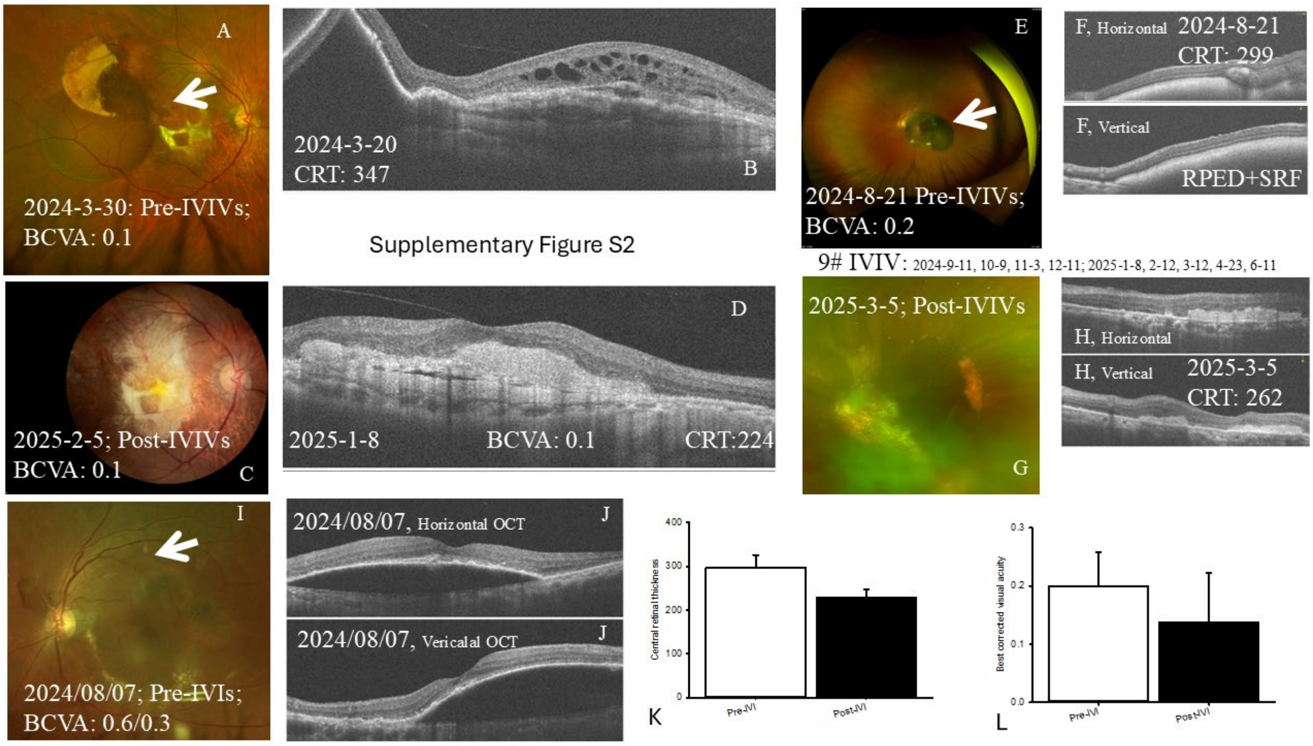

Supplementary Figure S2. PCV (n = 3) administered with fortified Vabysmo (12 mg/0.1 mL) for PCV with polyps. The research (Case 1: A~D; Case 2: E~H; Case 3: I~J) related to the patients with type C PCV revealed that the following anti-VEGF monotherapy, either fortified Avastin or fortified Vabysmo (IVIV), did not significantly decrease the central retinal thickness, CRT, nor did BCVA (Snellen E) significantly change. Two out of three eyes' PCV were dry and post-injection BCVAs were not improved due to fibrotic SRNVM as defined. Neither was the BCVA of the third eye that received 3 injections of anti-VEGF (IVI); the BCVA was not improved (from 0.3 to 0.3). Three cases of PCV with subretinal fluid (SRF) which was relieved by intravitreous injections of Vabysmo and/or Avastin with central retinal thickness, CRT, reduction, but not significant (K). However, BCVA was not improved (L). Abbreviations: polypoid choroidal vasculopathy (PCV), intravitreous injection of Vabysmo/Eylea/Lucentis (IVIV/IVIE/IVIL); best corrected visual acuity, BCVA; retinal pigment epithelium detachment, RPED; optical coherence tomography, OCT, including horizontal and vertical scans.

https://doi.org/10.3390/xxxxx

STROBE-compliant Flow Diagram

Intravitreal injections with aqueous humor collections for nvAMD/PCV/CSCR.

Patients assessed for eligibility and underwent intravitreal injection (n = 41/22/4)

— PCV that received PDT alone (Case 11) and rejected for signing the aqueous sample consent (Case 5 and 10).

Aqueous humor collected prior to injection (n = 41/19/4)

— Adequate sample volume obtained (n = 41/19/4)

— None aqueous collection performed (loosely attached PCV label loss) (n = 0/2/0)

Included in final analysis (n = 41/17/4)

Analyzed for aqueous cytokine / biomarker levels (as follows)

No of VEGF (AMD/PCV)=	41/	17	
No of PLGF (AMD/PCV)=	29/	13	
No of $HIF_{1\alpha}$ (AMD/PCV/CSCR)=	20/	5/	4
No of β-Catenin (AMD/PCV/CSCR)=	16/	9/	4
No of Wnt (PCV/CSCR)=		6/	4

Note: In the same follow-up period, we had more patients of AMD but less numerous type C PCV ones utilizing VEGF and PLGF to prove the difference between two identities. On the other hand, utilizing $HIF_{1\alpha}$, β-Catenin and/or Wnt, we tried to prove the similarity between PCV and CSCR with AMD as a control.

https://doi.org/10.3390/xxxxx
